# Human Placental Allograft Membranes: Promising Role in Cardiac Surgery and Repair

**DOI:** 10.3389/fcvm.2022.809960

**Published:** 2022-02-17

**Authors:** Pamela G. Hitscherich, Evangelia Chnari, Jessa Deckwa, Marc Long, Zain Khalpey

**Affiliations:** ^1^MTF Biologics, Edison, NJ, United States; ^2^Northwest Medical Center, Heart and Valve Institute, Cardiothoracic Surgery, Tucson, AZ, United States

**Keywords:** allograft, inflammation, cardiac surgery, heart disease, post-operative atrial fibrillation (POAF), placenta, amnion, membrane

## Abstract

Despite the immense investment in research devoted to cardiovascular diseases, mechanisms of progression and potential treatments, it remains one of the leading causes of death in the world. Cellular based strategies have been explored for decades, having mixed results, while more recently inflammation and its role in healing, regeneration and disease progression has taken center stage. Placental membranes are immune privileged tissues whose native function is acting as a protective barrier during fetal development, a state which fosters regeneration and healing. Their unique properties stem from a complex composition of extracellular matrix, growth factors and cytokines involved in cellular growth, survival, and inflammation modulation. Placental allograft membranes have been used successfully in complex wound applications but their potential in cardiac wounds has only begun to be explored. Although limited, pre-clinical studies demonstrated benefits when using placental membranes compared to other standard of care options for pericardial repair or infarct wound covering, facilitating cardiomyogenesis of stem cell populations *in vitro* and supporting functional performance *in vivo*. Early clinical evidence also suggested use of placental allograft membranes as a cardiac wound covering with the potential to mitigate the predominantly inflammatory environment such as pericarditis and prevention of new onset post-operative atrial fibrillation. Together, these studies demonstrate the promising translational potential of placental allograft membranes as post-surgical cardiac wound coverings. However, the small number of publications on this topic highlights the need for further studies to better understand how to support the safe and efficient use of placenta allograft membranes in cardiac surgery.

## Introduction

Cardiovascular diseases (CVD) remain a leading cause of death in the US and worldwide (1). Within CVD lie numerous conditions ranging from coronary artery disease (CAD) and myocardial infarction (MI) to stroke and heart failure (HF). Treatments range from lifestyle changes, medicinal management and surgery including coronary artery bypass and valve replacement. However, when interventions fail, heart transplant becomes the only option due to heart failure, the end stage condition for almost all CVDs (2). Due to donor shortages, heart transplant wait-times have increased in the last two decades which has limited transplant as a viable option for many patients (3).

One of the most common heart conditions is CAD, which can lead to MI. Every year millions of people suffer an MI (2) and experience irreversible damage to the myocardium leading to decreased functional capacity from loss of viable cardiomyocytes that cannot be replenished naturally (4). Over $300 billion was spent on the management of CVD in 2016, while indirect productivity costs associated totaled over $200 billion (5). Despite this, so much remains unknown about mechanisms that dictate the development and progression of CVD.

As a result, over $4 billion was spent on CVD research by the NIH alone in 2019 (6). One common area of focus is the post-MI microenvironment and how to prevent or repair the wounded tissue and consequent loss of viable tissue and function. Research in this area began with the idea of replacing or replenishing the lost cardiomyocytes because adult cardiomyocytes do not readily proliferate. Various cell sources were explored from transdifferentiated fibroblasts to stem cell derived populations (7), including placental derived cells such as amniotic epithelial (8–10) and stromal cells (11, 12).

However, there are mixed results in the field. Gorjipour et al. observed cell retention without functional improvement (13) while interestingly, some studies that found functional improvements post-MI did not require transplanted cell retention at all. In fact, Passipieri et al. demonstrated significant recovery of ejection fraction (EF) when transplanted placenta-derived mesenchymal stem cells remained in the tissue for only 4 days (14), suggesting the benefits of cellular based therapies are paracrine or soluble factor-based mechanisms.

This idea is reinforced by a recent study demonstrating conditioned media from human amniotic membrane derived mesenchymal stem cells alone significantly improved fractional shortening (FS) and EF, decreased apoptosis and fibrosis and significantly increased angiogenesis (15). The idea that cells themselves are not necessary to see improved heart function post-MI was also demonstrated by Vangnozzi et al. (16). Their group injected multiple types of viable stem cells, dead cells and zymosan, a chemical reagent into the hearts of post-ischemia/reperfusion mice. They found animals with all treatments experienced similar acute, sterile immune response governed by macrophages, improved cardiac fibroblast activity and the mechanical properties of the infarct with reduced fibrosis. These results suggest the critical role the immune system plays in regulating cardiac health and function after injury and contradicts the idea that cell-based therapies are necessary for cardiac improvement (17).

These recent pre-clinical findings shed new light on the strong relationship between CVD and inflammation. Although acute inflammation is necessary to respond to injury and disease and initiate healing of the wounded tissue, it is the overwhelming inflammation or the unnecessary delay in the transition from inflammatory to the proliferative/healing phase, that perpetuates injury in the heart causing larger scale functional deficits through advanced disease progression. Many factors have been explored to help coordinate the inflammatory response in the heart. Numerous drugs, including non-steroidal anti-inflammatory drugs have been used systemically to manage inflammation. However, further research is necessary to elucidate the true complexity of inflammation post-cardiac injury and how a more targeted approach may be more beneficial than a systemic treatment regimen (18).

**Figure 1:**
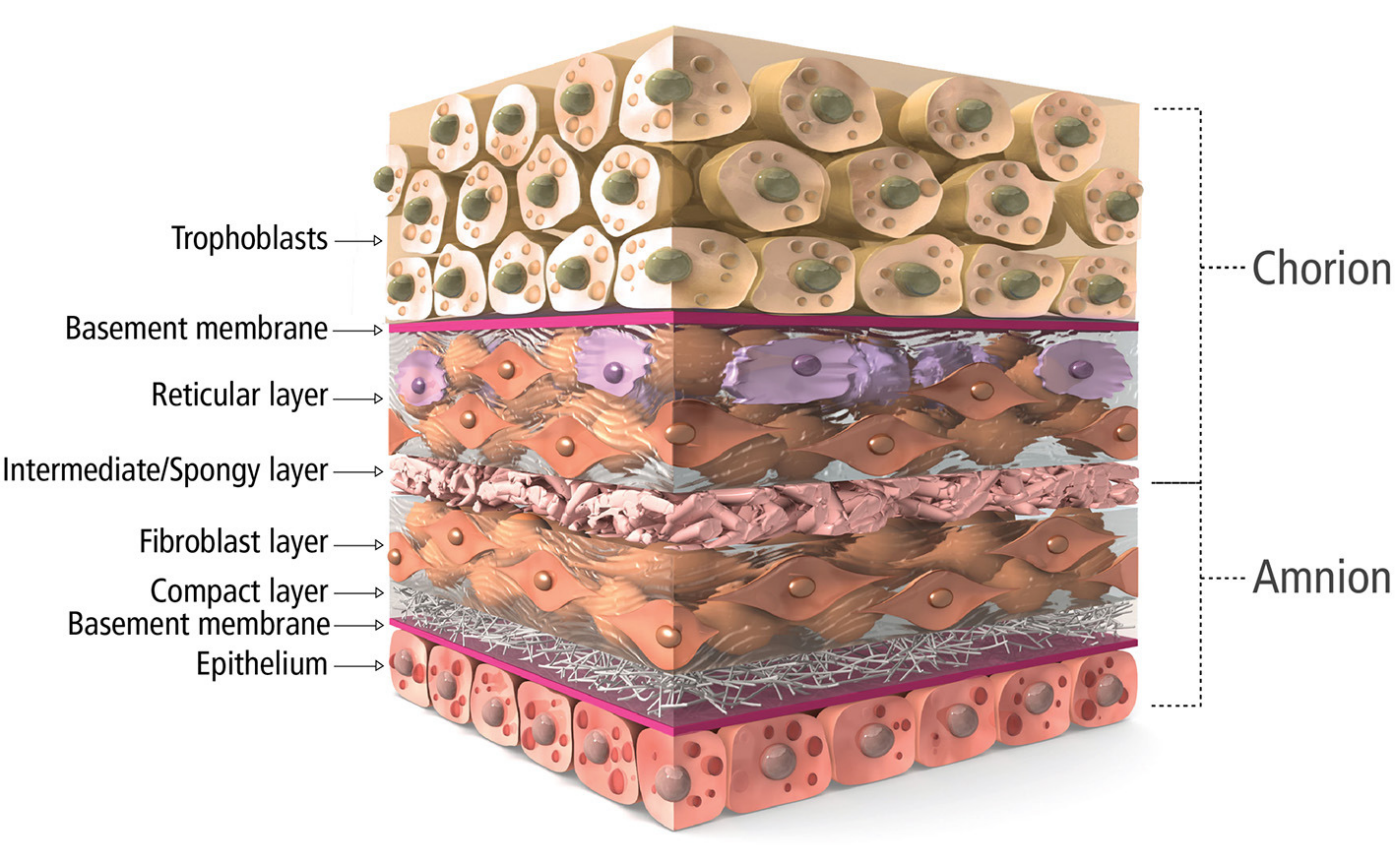
Cross-sectional schematic of placental tissue composition.

Along this line, placental tissue is known to be immune privileged, acting as the barrier during fetal development (19, 20). The placental membrane is a bilayer, amnion and chorion, composed of a complex, multilayer, collagen-rich extracellular matrix (Figure 1). Fibrillar collagens type I and III provide the mechanical strength and integrity while collagen V and VI help form connections to the basement membranes composed of collagen IV, fibronectin, and laminin (20, 21). Interspersed within these collagen networks are growth factors, cytokines, and chemokines with numerous functions. Growth factors such as TGF-α, TGF-β, PDGF and EGF regulate cell growth, proliferation, and survival while VEGF is known to be pro-angiogenic. Multiple inflammation-related proteins are also found in placental tissue including IL-1α, IL-1β, IL-10, IL-4, MCP-1 and tissue inhibitors of metalloproteinases, playing a role in balancing the needed acute inflammation while minimizing chronic, unmanaged inflammation (22–24). Because of these innate characteristics and components, placental allograft membranes (PAM) have been used successfully in ocular (25) and advanced wound care applications (26–30) supporting faster, more organized healing by covering the wound and offering matrix proteins and factors that may help guide the process. Because cardiac surgery and CVD including MI create wounds in the myocardial tissue, human PAM may offer similar benefits for this application as a covering of the wounded tissue. Some studies have demonstrated benefit with combining PAM with cardiac specific ECM (31), anti-inflammatory nanoparticles (32) and various cell types (33, 34), with some even demonstrating amniotic membrane culture promotes human iPSC-derived cardiomyogenesis (34, 35). However, we believe it is necessary to explore the past and current use of PAM on their own for cardiac surgery to better understand the science behind the potential benefits of using placenta tissue in another wound covering application and explore how processing of the tissue, including decellularization or terminal sterilization, may affect those benefits. We have conducted a thorough literature search in multiple databases for publications describing the use of placental tissue for heart disease applications. Out of over 200 references, 7 mention the use of intact, placental membrane used as a treatment for cardiac disease states and are described below and summarized in Table 1.

**Table 1:** Summary of pre-clinical and clinical studies of placental tissue in cardiac applications.

**Author**	**Tissue**	**Treatment**	**Timing**	**Technique**	**Application**	**Results**	**References**
Muralidharan et al.	Human Amnion	0.8% glutaraldehyde	2–5 days	Sutured to pericardium	Dog Pericardial substitute	• PTFE patches: • Moderate to severe lung/chest wall adhesions • Epicardial reactions • 2 with severe epicardial adhesions • AM patches: • Minimal lung/chest wall adhesions • No epicardial adhesions or reactions • Neovascularization	(36)
Cargnoni et al.	Human Amnion	Saline and antibiotics	24 h	Tied to ligation suture	Rat MI	• MI±Patch: • Improved LV Dimensions, EF, and FS up to 60 days • Smaller infarct up to 30 days • No difference at 90 days	(37)
Francisco et al.	Amnion	Sodium dodecyl sulfate	24 h	Sutured to pericardium	Rat Pericardial substitute	AAM: • Full incorporation and cell infiltration at 4 weeks • Thicker pericardial repair	(38)
Roy et al.	Human Amnion	• TGF-β1 (EMT) • 10 mM Tris/0.1% EDTA, 0.5% sodium dodecyl sulfate (decell)	• 7 days • 1 h, 4 h	Tied to ligation suture	Murine MI	Native AM: • Smaller infarct • CD4^+^ cell invasion • Decell AM: • Smaller infarct • Improved LV pressure, function, and wall thickness EMT AM: • CD4^+^ cell invasion	(39)
Lim et al.	Human Amnion/chorion	Dehydrated and terminally sterilized	-	sutured	Murine MI	dHACM: • Smaller infarct size • Higher c-kit^+^ cells • Higher proliferation and lower apoptosis • Higher CD31^+^ vessel density	(40)
Khalpey et al.	Human Amnion	Terminally sterilized	-	Topically Placed	Clinical Pericarditis, POAF	Patient with HAM: • Mild inflammation • No arrhythmia Patient without HAM: • Extensive inflammatory pericardial edema • NOPAF	(41)
Marsh et al.	Human Amnion	-	-	Topically Placed	Clinical Constrictive Pericarditis	Patient with HAM: • Limited pericardial constriction • No inflammation • No pericardial effusion	(42)

*PTFE, polytetrafluoroethylene; AM, amniotic membrane; LV, left ventricular; EF, ejection fraction; FS, fractional shortening; AAM, acellular amniotic membrane; dHACM, dehydrated human amnion/chorion bilayer membrane; HAM, human amniotic membrane; NOPAF, new onset post-operative atrial fibrillation*.

## Pre-Clinical

One of the first pre-clinical models exploring placental tissue for cardiac treatment by Cargnoni et al. investigated viable human amniotic membrane in both healthy and infarcted rats (37). MI+Patch rats demonstrated significantly better left ventricular (LV) dimensions in systole and wall thickness compared to MI-only rats up to 60 days. Differences were not influenced by degree of severity of the infarct, suggesting beneficial application for a wide array of pathologies. Additionally, MI+Patch rats had preserved LV function including EF, FS, and hemodynamic properties up to 60 days and a significantly smaller infarct scar up to 30 days. Interestingly, all significant differences associated with patch placement were no longer evident at 90 days post-surgery. Originally, Cargnoni et al. hypothesized that the benefits demonstrated were due to amniotic cell engraftment in the myocardium. However, no human cells were detected in rat hearts at both 30- and 90-days post-op, suggesting the beneficial effects could instead be due to the presence of soluble factors associated with inflammation, such as IL-6 and IL-10, from amniotic cells.

Dehydrated human amnion/chorion bilayer membrane (dHACM) application vs. saline injection on post-MI function was explored by Lim et al. in both wild type and NOD/SCID mice (40). At 8 weeks, mice with dHACM exhibited a significantly smaller infarct size. Interestingly, a significantly higher number of c-kit-positive cells were present in hearts with dHACM placement both at the site of treatment and remotely, suggesting homing of stem cell like cells to the site of injury. Additionally, significantly higher proliferation and lower apoptosis was evident in mice with dHACM along with significantly higher CD31 positive vessel density. Taken together, the results suggest dHACM patch placement may elicit paracrine pathways for enhanced autologous cell homing and increased blood supply leading to enhanced cardiac repair.

Roy et al. took it one step further and compared the potential placement of multiple forms of human amniotic membranes (AM) in a post-myocardial infarction (post-MI) BALB/c mouse model: native, decellularized (dAM) via lysis buffer and sodium dodecyl sulfate (SDS) or treated with TGF-β to induce epithelial-to-mesenchymal transition (EMT) in amniotic epithelial cells (39). Although Roy et al. previously reported enhanced cardioprotective capacity of amniotic endothelial cells after EMT (9), only hearts with native AM and dAM coverings exhibited smaller infarcts than MI-only hearts. Additionally, only hearts with dAM covering exhibited significantly improved pressure, function, and wall thickness. Conversely, EMT-AM and native AM covered hearts exhibited CD4+ cell invasion *in vivo* and *in vitro* EMT-AM promoted proliferation and activation of splenocytes and increased release of pro-inflammatory IL-6 from cultured cardiac fibroblasts. dAM released no IL-6 or IL-10, suggesting growth factors were removed during SDS decellularization, highlighting the importance of tissue processing methods during future therapy development. These results suggest that a cell free AM application may support ventricular function post-MI and that beneficial effects could be due more to ECM components than cellular derived growth factors, although further investigation is necessary.

While preclinical models focused mainly on the highly inflammatory post-MI microenvironment, other highly inflammatory conditions have also been explored. Many cardiac patients undergo reoperations, especially pediatric patients with congenital heart disease. Hence there is a need for a patch to replace or support the pericardium, aid in closure, and not elicit an uncontrolled inflammatory response or adhesion formation. Muralidharan et al. compared glutaraldehyde treated human AM and polytetrafluoroethylene (PTFE, Goretex) for pericardial closure in a mongrel dog model (36). After thoracotomy, multiple 3x4cm excisions were made and then patched with either a continuously sutured AM or PTFE membrane before closure. After 18 weeks, animals were sacrificed before undergoing a midline sternotomy to assess patch anatomy and adhesions. All PTFE patches showed moderate to severe lung/chest wall adhesions, while 2 dogs demonstrated marked epicardial reaction and severe epicardial adhesions at PTFE patch site causing epicardial hemorrhage and bleeding upon liberation. However, the AM patches placed to cover and protect the site of surgery demonstrated minimal lung/chest wall adhesions that were easily liberated and no epicardial adhesions or reactions. Additionally, histology showed AM patches remained intact with neovascularization and lymphatic infiltration while PTFE membranes induced fibroblast proliferation and infiltration. Taken together, Muralidharan et al. concluded AM placement as a pericardial supplement was superior to PTFE.

Similarly, Francisco et al. utilized a wistar rat model to explore the potential of using a human acellular amniotic membrane (AAM) to replace and support pericardial lesions (38). Specifically, the pericardium was excised from rats and either sutured back together or replaced with AAM sutured to the lesion. After 4 weeks, the AAM was fully incorporated into the tissue, as demonstrated by cell infiltration. Additionally, AAM placement supported a thicker pericardial repair suggesting not only that AAM is a safe alternative to other biomaterial substitutes but also supports better wound healing of pericardial lesions.

## Clinical

Early clinical use of placental membranes in cardiac applications is limited. Like Francisco et al., amniotic tissue enticed Khalpey et al. to explore the potential for decellularized human amniotic membrane (HAM) as a cardiac covering post cardiac surgery. Utilizing T2-weighted magnetic resonance imaging (MRI), with and without fat suppression, 2 patients undergoing cardiac surgery were analyzed (41). The first patient, a 56yo male with CAD requiring a third coronary artery bypass grafting (CABG) procedure, did not get a HAM covering. He exhibited extensive inflammatory pericardial edema post-surgery and developed new onset post-operative atrial fibrillation (NOPAF). NOPAF remains the most common complication of cardiac surgery occurring in over one third of open-heart surgical patients but is more than likely underestimated (41, 43, 44). Its management is difficult and requires short-term additional days in ICU/hospital and long-term use of anti-coagulants for stroke prevention. The second was a 24 yo male heart transplant patient suffering from constrictive pericarditis, treated with pericardial stripping, adhesiolysis and HAM placement to protect the heart tissue. Six days after surgery, patient 2 exhibited mild inflammatory pericardial edema and no arrhythmia. While this study presents only the beneficial clinical outcomes of one patient treated with HAM, the prolonged exploration of the hypothesis that HAM, used as a heart wound covering, may mediate post-surgical inflammation and decreased the propensity for NOPAF was presented in an abstract by Rao et al. (45).

Khalpey's group continued to explore the potential of placental tissue for cardiac wound and heart tissue covering applications. Specifically, Marsh et al. describes a complex case of constrictive pericarditis, a rare condition that inhibits diastolic filling due to a thickening and stiffening of the pericardium leading to myocardial ischemia (42). While the cause of pericarditis may vary, inflammation is a key component of the pathological process (46). The patient, a 34 yo male, was a heart transplant recipient with subsequent tricuspid valve repair due to severe regurgitation. He presented with severe fatigue and shortness of breath along with sinus tachycardia, ejection fraction <20% and Grade 1R mild acute rejection demonstrated via endomyocardial biopsy. Cardiac MRI revealed a thickened pericardium and evidence of fibrotic tissue along with tricuspid regurgitation. During surgery, the Gore-Tex membrane was found to be covered in a thick, gelatinous material and fused to the thickened pericardium, requiring removal, as well as a tricuspid valve replacement. In its place, 4 HAM allografts were topically applied to the anterior surface of the heart as wound coverings before closure to reconstruct the pericardium. Excised tissue analysis revealed chronic pericardial inflammation and fibrosis. After 5 weeks, although some constriction was still noted, T2-weighted MRI demonstrated no inflammation or pericardial effusion. Although these results are promising and suggest the use of HAM as a cardiac wound covering may mediate post-surgical inflammation, there is a need for further clinical evaluation including studies with higher patient numbers in order to determine the potential of PAM placement to support damaged and wounded heart tissue.

## Discussion

Our thorough review revealed a limited number of studies exploring the potential of PAM for cardiac applications despite its promising natural and intrinsic tissue properties. All pre-clinical studies demonstrated benefits when using PAM, either as a pericardial substitute or as a wound covering in the post-MI microenvironment. In one study, authors stated it offered benefits over widely used PTFE membrane for pericardial repair and promoted improved healing of pericardial lesions. In MI studies, multiple configurations of PAM demonstrated cardiomyogenesis of stem cell populations *in vitro* and supported functional performance *in vivo*. Most studies also discuss the hypothesis that the benefit of placental tissue may lie in its natural immunomodulatory properties, however further investigation is required to understand more fully the relationship between improved outcomes and allograft use. Further, the clinical studies presented demonstrate the translational potential of PAM as a cardiac wound covering in conditions where the heart tissue is damaged. These studies suggest the covering of the heart tissue wound may further mitigate the predominant inflammatory environment such as in the case of pericarditis and prevention of NOPAF, however clinical studies are very limited and further exploration must be done to fully elucidate the clinical potential of PAM.

Multiple placental components were investigated from amniotic membrane only, to an amnion/chorion bilayer as well as multiple preparations including preconditioning, decellularization and lyophilization. These configurations highlight the importance of considering tissue composition and processing for each application. Presented here was a wide range of processing techniques, from simple rinses or detergent decellularization to terminal sterilization and/or lyophilization. Each has advantages and disadvantages related to retention of native tissue matrix architecture and components, proteins and growth factors which should be considered thoroughly. Protocols that utilize multiple rinses may run the risk of washing away native growth factors, especially those that utilize strong detergents for decellularization, but retain native tissue architecture. While others that utilize terminal sterilization may retain more growth factors but cause changes to the ECM structure. Despite these differences, benefits were evident in all presented studies, suggesting it is not only one component that is of most importance, but a combination of factors working together to support tissue healing. A membrane comprising both amnion and chorion may provide benefits over amnion-only by increasing handling properties, as a thicker tissue bilayer, and an increased amount of naturally preserved components. It may also be suggested that aseptic processing without terminal sterilization may result in the highest retention of the natural architecture, components, and properties of human placental tissue, although more in-depth, pathway focused analysis of the effect of PAM are necessary to tease out the details.

In the clinic, considering the small number of publications on this topic, as highlighted in this review, further studies with defined and larger patient populations are required to better understand how to support the safe and efficient use of PAM in cardiac surgery with potential clinical benefits. Taken together, these studies demonstrate the promising translational potential of PAM as post-surgical cardiac wound coverings due to the natural properties and function of placental tissues. How the PAM is processed to retain its natural and inherent properties and components, but also how the membranes are applied during surgery to cover the cardiac wounds, may have an important impact on the clinical outcomes and patient recovery.

## Data Availability Statement

The original contributions presented in the study are included in the article/supplementary material, further inquiries can be directed to the corresponding author/s.

## Author Contributions

All authors listed have made a substantial, direct, and intellectual contribution to the work and approved it for publication.

## Conflict of Interest

ZK serves as a paid consultant for MTF Biologics. PH, EC, and ML were employed by MTF Biologics. The remaining author declares that the research was conducted in the absence of any commercial or financial relationships that could be construed as a potential conflict of interest.

## Publisher's Note

All claims expressed in this article are solely those of the authors and do not necessarily represent those of their affiliated organizations, or those of the publisher, the editors and the reviewers. Any product that may be evaluated in this article, or claim that may be made by its manufacturer, is not guaranteed or endorsed by the publisher.
